# Short- and Medium-Chain Fatty Acid Profiles in Biopsy-Proven Metabolic Dysfunction-Associated Steatotic Liver Disease: A Cross-Sectional Study

**DOI:** 10.3390/nu18213548

**Published:** 2026-10-28

**Authors:** Inmaculada Ros-Madrid, María A. Martínez-Sánchez, Rosario P. Cano-Mármol, Sara Rico-Chazarra, Lidia Sánchez-Alcoholado, Virginia E. Fernández-Ruiz, Alicia González-González, Carlos M. Martínez, Antonio Silva, Esteban N. Gurzov, Eliana Mariño, María D. Frutos, Mercedes Ferrer-Gómez, José C. Fernández-García, María Á. Núñez-Sánchez, Bruno Ramos-Molina

**Affiliations:** 1 Obesity, Diabetes and Metabolism Laboratory, Biomedical Research Institute of Murcia (IMIB), 30120 Murcia, Spain; inmarosmadrid@gmail.com (I.R.-M.); mariaantonia.martinez1@gmail.com (M.A.M.-S.); palomacanomarmol96@gmail.com (R.P.C.-M.); sara.ricoc@um.es (S.R.-C.); l.s.alcoholado@gmail.com (L.S.-A.); virginiaesperanza.fernandez@um.es (V.E.F.-R.); tofly16@gmail.com (M.F.-G.); 2 Department of Endocrinology and Nutrition, Virgen de la Arrixaca University Hospital, 30120 Murcia, Spain; 3 Department of Endocrinology and Nutrition, Regional University Hospital of Málaga, Biomedical Research Institute of Málaga and Nanomedicine Platform (IBIMA-Plataforma BIONAND), 29010 Málaga, Spain; agonzalez.bq@gmail.com; 4 Department of Anatomy and Comparative Pathology, Faculty of Veterinary Science, University of Murcia, Campus de Espinardo, 30100 Murcia, Spain; cmmarti@um.es; 5 Facility of Innovation and Analysis in Animal Source Foodstuffs of SAIUEx, University of Extremadura, 10003 Cáceres, Spain; asilvaro@unex.es; 6 Signal Transduction and Metabolism Laboratory, Université libre de Bruxelles, 1070 Brussels, Belgium; esteban.gurzov@ulb.be; 7 ImmunoBiota Therapeutics Pty Ltd., Melbourne 3187, Australia; eliana.marino@immunobiota.com; 8 Department of General and Digestive System Surgery, Virgen de la Arrixaca University Hospital, 30120 Murcia, Spain; mfb25s@gmail.com; 9 Centro de Investigación Biomédica en Red de Diabetes y Enfermedades Metabólicas Asociadas (CIBERDEM), Instituto de Salud Carlos III, 28029 Madrid, Spain; 10 Department of Medicine and Dermatology, Faculty of Medicine, University of Malaga, 29071 Malaga, Spain

**Keywords:** short-chain fatty acids, metabolic dysfunction-associated steatotic liver disease, metabolic dysfunction-associated steatohepatitis, obesity, acetate

## Abstract

Background/Objectives: Metabolic dysfunction-associated steatotic liver disease (MASLD) is a common metabolic liver disease with increasing prevalence worldwide. Increasing evidence suggests that short-chain fatty acids (SCFAs) play an important role in MASLD. Medium-chain fatty acids (MCFAs), although less studied, may also contribute to MASLD pathogenesis. The aim of this study was to evaluate plasma SCFA and MCFA profiles across the MASLD histological spectrum and their associations with clinical, biochemical, and histopathological features of disease severity. Methods: We included 115 adults with obesity undergoing bariatric surgery. Intraoperative liver biopsies were assessed using the Steatosis, Activity, Fibrosis (SAF) score. Participants were classified as no MASLD (*n* = 26), metabolic dysfunction-associated steatotic liver (MASL; *n* = 42), or metabolic dysfunction-associated steatohepatitis (MASH; *n* = 47). Six SCFAs and two MCFAs were quantified in plasma by liquid chromatography–mass spectrometry. Results: Patients with MASH showed lower acetate and higher hexanoate plasma levels than those without MASLD. Acetate was inversely associated with ALT, AST, GGT, glucose, HbA1c, cytokeratin-18, SAF score, steatosis and lobular inflammation, whereas hexanoate was positively associated with SAF score and lobular inflammation. Higher acetate concentrations were independently associated with lower odds of MASH (OR 0.973; *p* = 0.040), while ALT levels were independently associated with increased odds of MASH (OR 1.083, *p* = 0.001). Conclusions: MASH in patients with obesity is characterized by a distinct circulating fatty acid profile, with reduced acetate and increased hexanoate. Lower acetate levels were independently associated with MASH and markers of metabolic and histological disease severity.

## 1. Introduction

Metabolic dysfunction-associated steatotic liver disease (MASLD) currently affects approximately 38% of adults and 7–14% of children and adolescents, with projections suggesting that by 2040 over 55% of adults may be affected by this disease [1]. MASLD is diagnosed based on evidence of hepatic steatosis combined with at least one metabolic risk factor in the absence of secondary causes of steatosis [1]. In the pathophysiology of MASLD, coexisting metabolic conditions such as type 2 diabetes mellitus (T2DM) can accelerate disease progression. Importantly, a proportion of patients with MASLD may progress to metabolic dysfunction-associated steatohepatitis (MASH), and in some cases, to advanced fibrosis, cirrhosis, and hepatocellular carcinoma [1,2,3].

The pathogenetic processes of MASLD involve sustained overnutrition, adipose tissue dysfunction, and the development of insulin resistance. These alterations promote hepatic lipogenesis and the accumulation of lipotoxic intermediates, triggering cellular stress responses within the liver. Concomitantly, disturbances in gut microbiota composition and immune homeostasis further exacerbate hepatic injury [3]. Growing evidence indicates that gut dysbiosis contributes to MASLD by altering the production of microbial metabolites such as short-chain fatty acids (SCFAs), bile acids, amino acid-derived metabolites and ethanol [4,5]. Nevertheless, the precise mechanisms linking gut dysbiosis to MASLD pathogenesis remain incompletely understood.

SCFAs, primarily acetate, propionate, and butyrate, are microbial metabolites largely generated through the intestinal fermentation of dietary fiber [6]. They have been described to exert beneficial effects on MASLD, partly through the activation of AMP-activated protein kinase (AMPK), a central regulator of hepatic metabolism [7]. This activation enhances insulin sensitivity, suppresses gluconeogenesis, and limits lipogenesis [7]. Within the pancreas, SCFAs are able to stimulate insulin release, support β-cell proliferation, and attenuate inflammatory responses, thereby contributing to the prevention of T2DM [8]. In adipose tissue, they alleviate inflammation and promote reductions in fat mass by enhancing lipolysis and β-oxidation, ultimately aiding in the management of obesity [8]. Moreover, SCFAs have been linked with the production of peptide YY and glucagon-like peptide 1 (GLP-1) by L cells in the intestine, which may influence satiety and energy intake [9].

On the other hand, medium-chain fatty acids (MCFAs) are saturated fatty acids generally containing six to twelve carbon atoms and differ from long-chain fatty acids in their absorption and hepatic metabolism [10]. Dietary MCFAs are absorbed directly into the portal circulation and are rapidly transported to the liver, where they undergo mitochondrial β-oxidation without requiring the carnitine palmitoyltransferase system as a rate-limiting step for the mitochondrial oxidation of long-chain fatty acids [11,12]. Beyond their role as energy substrates, increasing evidence suggests that MCFAs may also act as signaling molecules and influence metabolic and inflammatory pathways involved in hepatic lipid metabolism. In particular, some MCFAs can activate G protein-coupled receptor 84 (GPR84), a free fatty acid receptor predominantly expressed in immune cells, thereby linking MCFA signaling to inflammatory responses [11,13,14]. Although modulation of GPR84 has been proposed as a potential therapeutic strategy in MASH, the role of endogenous circulating MCFAs in human MASLD remains poorly characterized.

Despite growing evidence implicating SCFAs in metabolic and inflammatory pathways relevant to MASLD, to date, most studies have been conducted in experimental models and have yielded inconsistent results, while evidence from human studies remains limited. Furthermore, even less is known about MCFAs and their potential involvement in MASLD. Importantly, whether circulating SCFA and MCFA levels differ across the histological spectrum of MASLD in individuals with obesity and relate to disease severity remains unclear.

Therefore, in this study we aimed to characterize circulating SCFA and MCFA levels in individuals with obesity and biopsy-proven MASLD, and to investigate their associations with clinical, biochemical and histopathological features of disease severity.

## 2. Materials and Methods

### 2.1. Study Participants

This study included 123 consecutive adults with obesity who underwent bariatric surgery at Virgen de la Arrixaca University Hospital (Murcia, Spain) between January 2020 and October 2022. Eligible participants were aged 18–65 years and had either a body mass index (BMI) ≥ 40 kg/m^2^ or a BMI ≥ 35 kg/m^2^ in the presence of clinically relevant obesity-related comorbidities (T2DM, uncontrolled hypertension, or obstructive sleep apnea syndrome), and a documented history of obesity lasting at least 5 years. T2DM was defined by fulfilling at least one of the modified diagnostic criteria of the International Diabetes Federation (IDF) and the World Health Organization (WHO) [15].

Exclusion criteria for this study were chronic liver disease of any etiology other than MASLD, excessive alcohol consumption (>30 g/day in men or >20 g/day in women), current use of steatogenic medications or GLP-1 receptor agonists, or refusal to provide informed consent. Of the 123 patients initially assessed for eligibility, 5 were excluded because of using GLP-1 agonist and 3 due to excessive alcohol consumption, resulting in a final study population of 115 patients.

Missing data were not imputed. Patients with missing data for a given variable were excluded only from the analyses involving that variable (complete-case analysis).

All participants underwent intraoperative liver biopsy during bariatric surgery, allowing histological assessment of MASLD and its severity. Based on the liver histology, patients were categorized into three subgroups, as previously described [16]: (1) no MASLD; (2) MASL, defined as the presence of ≥5% hepatic steatosis (grade 1 or higher) with either hepatocellular ballooning or lobular inflammation, but not both simultaneously; and (3) MASH, characterized by steatosis grade 1 or higher together with ballooning and lobular inflammation, with or without fibrosis.

The study was carried out in accordance with the Declaration of Helsinki and local regulations. Ethical approval was obtained from the Ethics and Clinical Research Committee of Virgen de la Arrixaca University Hospital (approval code 2020-2-4-HCUVA, 31 March 2020), and all participants provided written informed consent prior to enrollment.

### 2.2. Clinical and Biochemical Parameters

Participants completed a structured preoperative clinical evaluation to obtain the following data: age, medical history, current diseases, and associated treatment. In addition, anthropometric parameters, including body weight, height, and waist circumference, were recorded. Body mass index (BMI) was calculated according to Quetelet’s formula:

BMI = weight (kg)/height^2^ (m^2^).

Body composition was assessed using a 50 kHz bioelectrical impedance analyzer (Tanita MC-980MA-N plus, Tanita Inc., Amsterdam, The Netherlands), according to the manufacturer’s instructions.

Preoperative biochemical data were also collected on the day of bariatric surgery after a minimum fasting period of 12 h. Blood samples were centrifuged, and serum was used for subsequent analyses. Standard laboratory determinations included fasting glucose, total cholesterol (TC), high-density lipoprotein cholesterol (HDLc), triglycerides (TG), alanine aminotransferase (ALT), aspartate aminotransferase (AST), gamma-glutamyl transferase (GGT), apolipoprotein A-I (Apo-AI), and apolipoprotein B (Apo-B), all measured using the Cobas c702 analyzer (Roche Diagnostics, Sant Cugat del Vallès, Spain) following standardized protocols. Low-density lipoprotein cholesterol (LDLc) was estimated using the Friedewald equation [LDLc = TC − HDLc − (TG/5)]. Glycated hemoglobin (HbA1c) was quantified with the Tosoh HLC-723G8 glycohemoglobin analyzer (Tosoh Bioscience, Tessenderlo, Belgium). Serum insulin levels were determined with the Cobas e801 analyzer (Roche Diagnostics). Insulin resistance was assessed using the Homeostasis Model Assessment for Insulin Resistance (HOMA-IR), calculated as [insulin (µU/mL) × glucose (mmol/L)]/22.5 [17].

Cytokeratin-18 (CK-18) M30 levels were determined in serum samples using the M30 Apoptosense^®^ ELISA kit (PEVIVA, VLVbio AB, Nacka, Sweden) according to the manufacturer’s instructions. Absorbance was determined at 450 nm using the Spectrostar Nano microplate reader (BMG LABTECH, Ortenberg, Germany) and CK-18 M30 levels (U/L) were calculated using the corresponding standard curve.

### 2.3. Liver Biopsies Collection and Sample Processing

During bariatric surgery, liver tissue samples of at least 1 cm^2^ were collected. Liver biopsies were fixed in 4% neutral buffered formalin (pH 7.4), and paraffin-embedded. Histological analyses were carried out on 5 µm paraffin sections stained with hematoxylin and eosin, Masson’s trichrome, periodic acid–Schiff, Prussian blue, and reticulin. All biopsy specimens were independently reviewed and scored by experienced liver pathologists at Virgen de la Arrixaca University Hospital and the Experimental Pathology Unit of the Biomedical Research Institute of Murcia, according to the Steatosis, Activity, Fibrosis (SAF) scoring system [18]. Pathologists were blinded to the participants’ clinical, biochemical, and metabolomic data.

### 2.4. Analysis of SCFAs and MCFAs in Plasma Samples by LC-MS

Six SCFAs and two MCFAs were quantified in plasma samples using liquid chromatography–mass spectrometry (LC-MS). The SCFA analyzed were acetate (C2:0), propionate (C3:0), butyrate (C4:0), isobutyrate (C4:0), valerate (C5:0), and isovalerate (C5:0), whereas the MCFAs analyzed were caproate (hexanoate; C6:0) and heptanoate (C7:0). All analytical standards were obtained from Sigma-Aldrich (St. Louis, MO, USA).

Sample preparation and LC–MS analysis was performed as previously described [19]. Briefly, 100 μL of plasma was mixed with 100 μL of methanol to precipitate proteins. After shaking for 1 min, samples were centrifuged at 14,000 rpm for 10 min at 4 °C. Subsequently, 100 μL of the supernatant was derivatized using 30 μL of 0.1 M O-benzylhydroxylamine hydrochloride in methanol and 30 μL of 0.25 M *N*-(3-dimethylaminopropyl)-*N*′-ethylcarbodiimide hydrochloride in methanol. The reaction was incubated at 25 °C for 1 h. The derivatized samples were then extracted with dichloromethane, which was subsequently evaporated. The resulting residue was reconstituted in 250 μL of methanol–water before injection.

Chromatographic separation was performed using an Infinity II UHPLC system (Agilent Technologies, Santa Clara, CA, USA) equipped with a reversed-phase Poroshell C18 EC column (100 × 2.1 mm, 3 μm; Agilent Technologies) maintained at 35 °C. The injection volume was 4 μL, and the mobile phase was delivered at a flow rate of 0.4 mL/min using solvent A (0.2% formic acid in water) and solvent B (0.2% formic acid in methanol). Gradient elution was performed with 15%, 15%, 70%, and 98% solvent B at 0, 3, 8, and 15 min, respectively. Detection was performed using an Agilent Technologies 6470 triple-quadrupole mass spectrometer equipped with an electrospray ionization (ESI) source operating in positive-ion mode. The source conditions were as follows: capillary voltage, 3.0 kV; source temperature, 200 °C; source gas flow, 10 L/min; nebulizer pressure, 20 psi; and sheath gas temperature and flow, 250 °C and 10 L/min, respectively. Collision energies were optimized within a range of 5–30 eV. Multiple reaction monitoring (MRM) transitions were optimized by injecting 1 ppm solutions of the individual fatty acid standards to select the transitions providing the best sensitivity and selectivity. Quantification was based on matrix-matched calibration using plasma samples spiked with a standard mixture at five concentrations (0.25, 0.5, 1, 5, and 10 μM). This approach enabled the evaluation of matrix effects and analyte recovery within the analytical procedure, as previously described [19].

### 2.5. Statistical Analysis

Results are presented as median (interquartile range). Normal distribution of the study variables was assessed using the Kolmogorov–Smirnov test. Group comparisons were conducted using the Kruskal–Wallis test, followed by post hoc pairwise analyses with the Mann–Whitney U test. To account for multiple testing, the Bonferroni correction was applied, setting the significance threshold at *p* < 0.0167 (0.05/3 comparisons). For the subgroup analysis assessing the influence of T2DM among participants with established MASLD, participants with T2DM but without MASLD were excluded. Categorical variables were compared using the chi-square test, and associations between continuous variables were evaluated using Spearman’s rank correlation test. Univariate logistic regression analyses were initially performed to identify demographic, anthropometric, clinical, and biochemical factors associated with the presence of MASH. Multicollinearity was assessed using variance inflation factor (VIF) and tolerance values. Sex, age and variables with *p* < 0.10 in univariate logistic regression analysis were considered eligible for inclusion in the multivariable model. A backward stepwise variable selection procedure based on the Wald statistic was used, with a significance criterion of *p* < 0.05 for entry and *p* > 0.10 for removal. Statistical analyses were performed using SPSS version 28.0.1.1 (IBM Corp., Armonk, NY, USA). Figures were generated with Prism version 9.0 (GraphPad Software, La Jolla, CA, USA).

## 3. Results

### 3.1. Characteristics of the Study Population

A total of 115 patients were included in this study, of whom 23% (*n* = 26) had no MASLD, 37% (*n* = 42) had MASL and 40% (*n* = 47) had MASH. Detailed clinical and biochemical characteristics of the study population are presented in Table 1.

The mean age of the study population was 47.3 years; the mean BMI was 42.9 kg/m^2^, and women predominated across all groups. Prediabetes and T2DM were present in 39% (*n* = 45) and 31% (*n* = 36) of patients, respectively, with a higher prevalence observed among patients with more advanced liver disease. Accordingly, fasting glucose, HbA1c, insulin, and HOMA-IR were higher in patients with MASL and MASH. The lipid profile was characterized by elevated triglyceride and Apo-B levels and lower HDL-C, Apo-AI concentrations in patients with MASLD. Liver injury markers, including transaminases and CK-18, were also higher in patients with MASL and MASH.

Table 2 summarizes the histological features of liver biopsies in patients with MASLD. The SAF score, which integrates steatosis, activity (hepatocellular ballooning and inflammation), and fibrosis, was significantly higher in patients with MASH compared to those with MASL. Analysis of individual components showed that steatosis grade differed significantly between groups, with higher grades in patients with MASH.

### 3.2. Differences in Plasma SCFAs and MCFAs Depended on the Presence of MASLD

**Figure 1:**
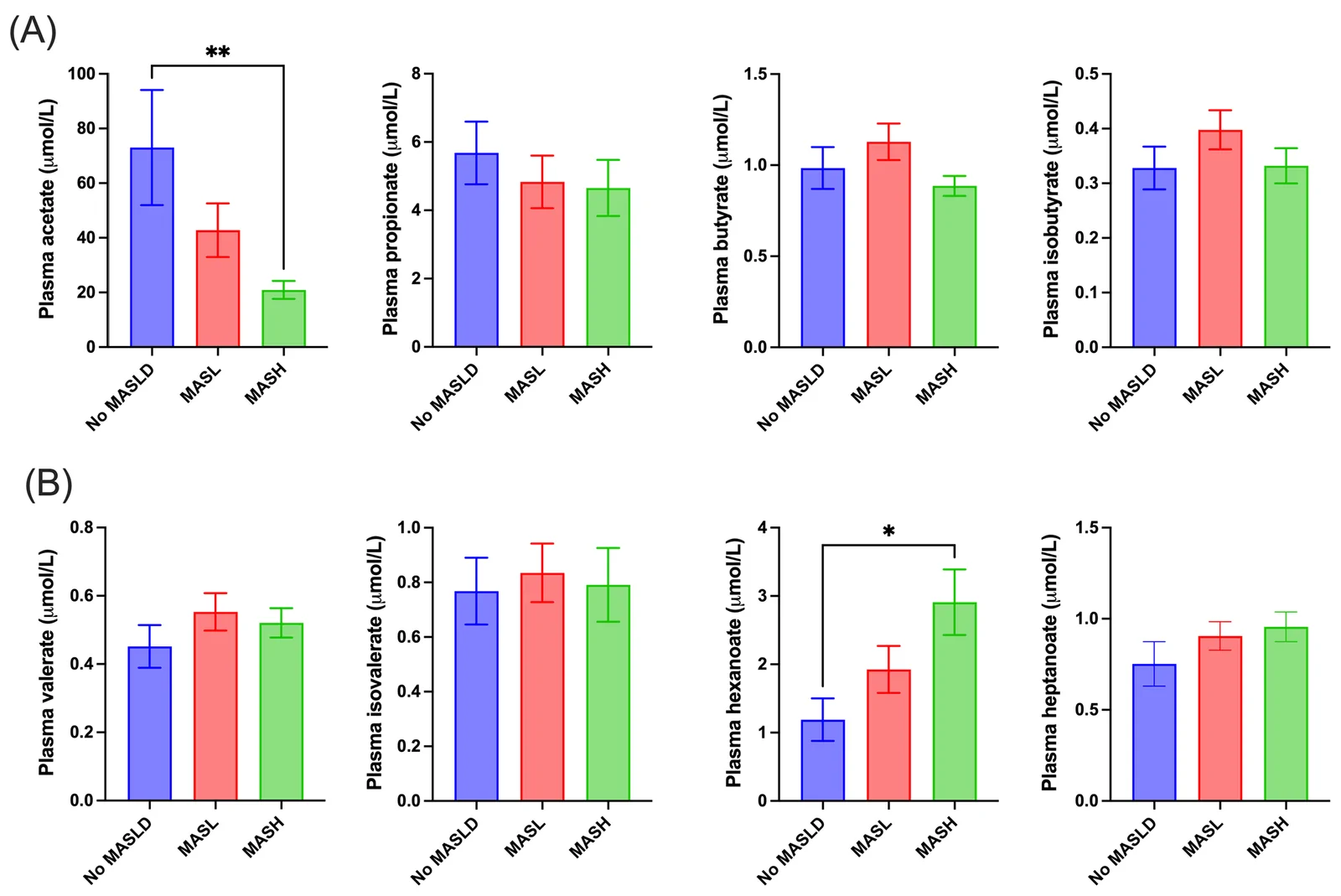
Plasma levels of SCFAs (**A**) and MCFAs (**B**) according to MASLD severity. Statistical differences were assessed using the Kruskal–Wallis test followed by post hoc pairwise analyses with the Mann–Whitney U test. To account for multiple testing, the Bonferroni correction was applied, setting the significance threshold at *p* < 0.0167. Significance levels are indicated as follows: * *p* < 0.05, ** *p* < 0.01. MASH, metabolic dysfunction–associated steatohepatitis; MASL, metabolic dysfunction–associated steatotic liver; MASLD, metabolic dysfunction-associated steatotic liver disease.

Among the measured species, acetate was the most abundant, followed by propionate. Compared with individuals without MASLD, patients with MASH exhibited lower plasma acetate levels and higher hexanoate concentrations (Figure 1). No significant differences were observed in the plasma concentrations of the remaining SCFA and MCFA species analyzed between the study groups.

**Figure 2:**
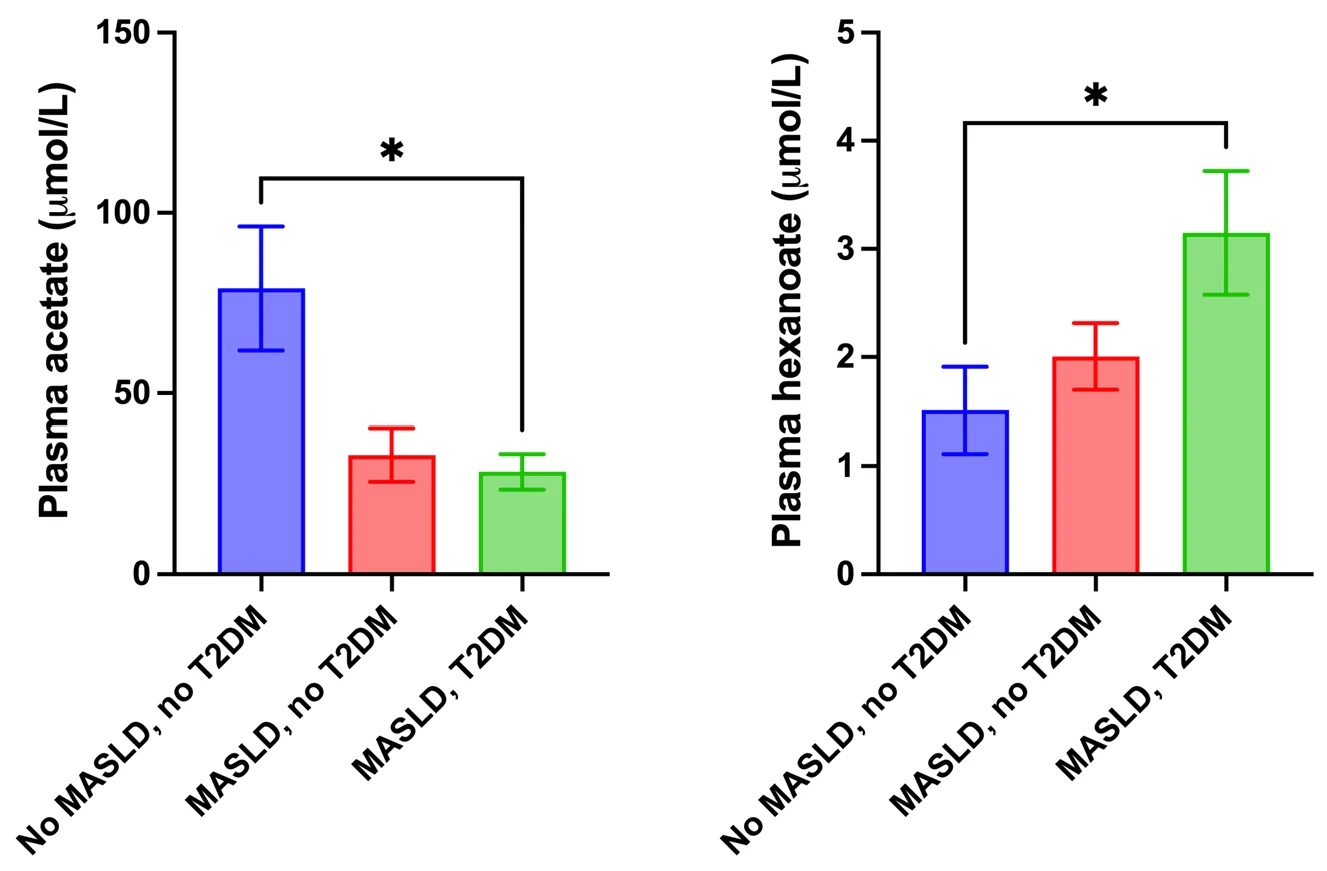
Plasma acetate and hexanoate levels according to liver pathology and T2DM status. Groups: No MASLD, no T2DM (*n* = 15), MASLD, no T2DM (*n* = 55), MASLD, T2DM (*n* = 34). Statistical differences were assessed using the Kruskal–Wallis test followed by post hoc pairwise analyses with the Mann–Whitney U test. To account for multiple testing, the Bonferroni correction was applied, setting the significance threshold at *p* < 0.0167. Significance levels are indicated as follows: * *p* < 0.05. T2DM, type 2 diabetes mellitus; MASLD, metabolic dysfunction-associated steatosis liver disease.

No differences were found in the plasma levels of the other species analyzed between study groups. When patients were stratified according to both MASLD and T2DM status, individuals with MASLD and T2DM showed lower plasma acetate and higher hexanoate levels than those without MASLD and without T2DM. However, among patients with MASLD, plasma SCFA and MCFA concentrations did not differ according to T2DM status (Figure 2).

### 3.3. Correlations Between Plasma SCFAs/MCFAs and Histopathological/Biochemical Parameters Related to MASLD

**Figure 3:**
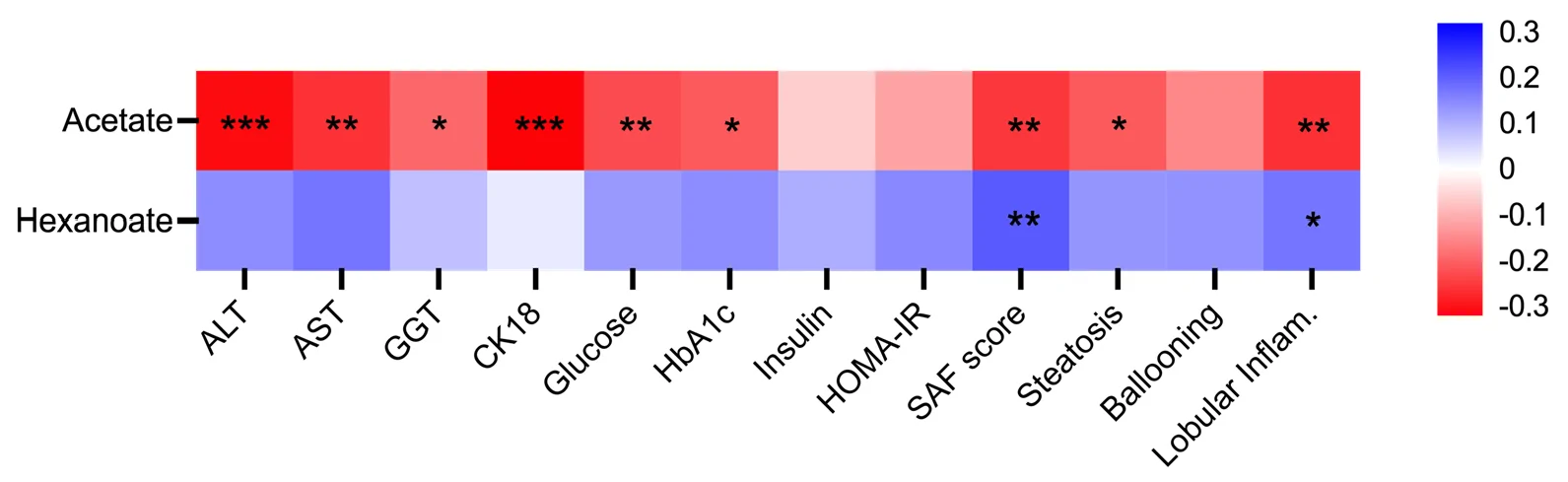
Correlations between acetate/hexanoate and biochemical and histopathological parameters related to MASLD. Values are presented as Spearman correlation coefficients. Significance levels are indicated as follows: * *p* < 0.05; ** *p* < 0.01; *** *p* < 0.001. Abbreviations: ALT, alanine aminotransferase; AST, aspartate aminotransferase; CK-18, cytokeratin 18; GGT, gamma-glutamyl transferase; HbA1c, glycated hemoglobin; HOMA-IR, homeostatic model assessment of insulin resistance.

Correlation analyses were performed to assess the relationship between plasma levels of acetate and hexanoate and the histopathological and biochemical parameters significantly altered in patients with MASLD (Figure 3). Our results showed that acetate exhibited an inverse correlation with ALT (r: −0.309; *p* < 0.001) and CK-18 (r: −0.320; *p* < 0.001), AST (r: −0.260; *p* = 0.003), GGT (r: −0.193; *p* = 0.003), glucose (r: −0.228; *p* = 0.009), HbA1c (r: −0.212; *p* = 0.009), SAF score (r: −0.248; *p* = 0.004), steatosis (r: −0.212; *p* = 0.015), and lobular inflammation (r: −0.261; *p* = 0.003). In contrast, plasma hexanoate levels displayed a weak positive correlation with both SAF score (r: 0.184, *p* = 0.036) and lobular inflammation (r: 0.172, *p* = 0.050).

### 3.4. Factors Associated with MASH

To identify factors associated with MASH, a univariate logistic regression analysis was initially performed. In this analysis, T2DM, HbA1c, HOMA-IR, and ALT levels were associated with the presence of MASH (Supplementary Table S1).

Next, a parsimonious multivariate logistic regression model was constructed. Of the 115 patients included in the study, 100 were included in the multivariable logistic regression analysis after excluding 15 patients because of missing data. Among these 100 patients, 36 had MASH. We identified that higher plasma acetate concentrations were associated with lower odds of prevalent MASH (OR 0.973, 95% CI 0.947–0.999, *p* = 0.040), indicating that each one-unit increase in plasma acetate was associated with approximately 2.7% reduction in the odds of having MASH. Conversely, ALT levels were independently associated with the presence of MASH (OR 1.083, 95% CI 1.035–1.133, *p* = 0.001). HOMA-IR was not independently associated with MASH after adjustment (OR, 1.176; 95% CI, 0.979–1.412; *p* = 0.083) (Table 3).

## 4. Discussion

In the present study, we evaluated plasma SCFA profiles across different stages of MASLD in patients with obesity. Our findings identified a distinct plasma fatty acid profile associated with MASLD severity, characterized by reduced acetate and increased hexanoate levels specifically in patients with MASH. Notably, these alterations were observed exclusively in patients with MASH, suggesting that changes in circulating fatty acid metabolism may be particularly associated with the more advanced and inflammatory phenotype of MASLD. In addition, multivariable logistic regression analysis identified higher plasma acetate concentrations as independently associated with lower odds of prevalent MASH, whereas higher ALT levels were independently associated with increased odds of MASH.

The lower acetate levels observed in our cohort are consistent with the findings reported by Thing et al. [20], who described lower plasma acetate levels in patients with MASLD compared with healthy individuals, as well as increases in propionate, formate, valerate, and methylbutyrate, which were associated with a higher degree of fibrosis. In contrast, Tsai et al. [21] reported decreases in formate, isobutyrate, and methylbutyrate levels, together with an increase in isovalerate. Notably, all participants in the study by Tsai et al. [21] had T2DM, whereas only 31% of our cohort had T2DM. In addition, our population consisted exclusively of individuals with obesity who were stratified according to the presence and severity of MASLD. Differences in study design may partly account for the discrepancies in SCFA profiles observed between studies.

Although we did not observe differences in plasma propionate levels according to the presence of MASLD in our cohort, preclinical studies have reported that supplementation with this SCFA led to reductions in body weight, blood pressure, insulin levels, hepatic steatosis, and liver inflammation in mice [22]. Moreover, propionate has been linked to greater weight loss following bariatric surgery [19]. The absence of significant differences in our cohort may reflect differences in study population, dietary intake, gut microbiota composition, medication use, or concomitant metabolic conditions, which could partly explain the discrepancies between experimental and human studies.

Butyrate is a SCFA that suppresses the immune response and reduces liver inflammation through inhibition of histone deacetylases [23]. Previous studies have reported increased levels in MASL and decreased levels in MASH [24], while in our cohort we observed only a slight downward trend (*p* = 0.059). In addition, Xiong et al. [24] observed a significative reduction in butyrate levels in patients with cirrhosis. Similarly to previous studies reporting no significant differences in circulating butyrate between patients with MASLD and healthy controls [19], we did not observe significant variations in circulating butyrate levels in our cohort. This discrepancy may reflect differences in study design, population characteristics, or metabolic context, such as the elevated BMI (>40 kg/m^2^) in our cohort. Importantly, accumulating evidence indicates that, among the different SCFAs, acetate exerts the most pronounced effect on reducing hepatic steatosis compared with butyrate and propionate [25].

Acetate is the most abundant SCFA in circulation, accounting for about 60% of total SCFAs [7], and therefore predominates in serum compared with propionate and butyrate [26]. In our study, we specifically observed reduced plasma acetate levels in patients with MASH. Furthermore, multivariate analysis showed that low acetate levels were independently associated with the presence of MASH, together with elevated ALT. Acetate levels were significantly lower in patients with MASH than in those without MASLD, whereas no significant differences were observed between the MASL and no-MASLD groups. Similarly, other studies have reported no differences in circulating acetate between with and without MASLD, whereas acetate concentrations decreased in patients with cirrhosis compared with those with MASLD [26]. The higher acetate levels observed in earlier stages of disease may reflect a compensatory response to inflammation or a secondary consequence of progressive alterations in the gut microbiota during MASLD progression [24].

In our study, while T2DM did not significantly alter acetate levels in patients with MASLD, poor glycemic control was associated with lower acetate concentrations. This finding suggests that glycemic control may influence circulating acetate levels, although the underlying mechanisms remain to be established. Experimental evidence from a diabetic mouse model supports this notion as acetate supplementation led to lower fasting plasma glucose and HbA1c levels compared with controls [27]. These effects were mediated, at least in part, by activation of hepatic AMPK, an energy-sensing enzyme that plays a pivotal role in glucose homeostasis. AMPK regulates multiple metabolic pathways, including the expression of glucose transporter type 4 (GLUT4), while enhancing fatty acid oxidation and reducing lipogenesis [25]. Furthermore, circulating acetate levels have been inversely associated with ALT and AST levels, in line with our results showing negative correlation between acetate concentrations and these liver enzymes [25]. Previous preclinical studies have demonstrated that acetate supplementation reduces hepatic lipid accumulation and mitigates pro-inflammatory macrophage activity and TNF-α expression [24,25]. Taken together, these findings support further investigation of acetate metabolism as a potential therapeutic target in MASLD, while acknowledging that the clinical relevance of these experimental effects remains to be established.

Regarding hexanoate, circulating levels were generally low, and although an increase was observed in patients with MASH. Furthermore, although hexanoate levels correlated with the SAF score, no significant correlations with other biochemical or histopathological variables were identified. This may be explained by its low concentrations and the limited sample size. In line with our findings, an in vitro study using HepG2 cells reported that hexanoic acid, compared with long-chain fatty acids, did not promote hepatic lipid accumulation or the development of insulin resistance [28]. Furthermore, in a preclinical study, supplementation with hexanoate at 2.5% of total dietary weight for 12 weeks did not show significant effects on liver steatosis or inflammation [22]. Taken together, these data suggest that, unlike acetate, the association of circulating hexanoate with MASLD may be less pronounced and its potential pathophysiological relevance remains unclear.

Our study has certain limitations that should be acknowledged. First, the relatively small sample size may have limited statistical power to detect weaker associations, particularly for metabolites present at low circulating concentrations. Second, the cross-sectional design of the analysis precludes establishing causal relationships between circulating SCFAs/MCFAs and MASLD severity. Third, the cohort was predominantly female and consisted of individuals with severe obesity undergoing bariatric surgery, which may limit the generalizability of our findings to other populations. In addition, the limited representation of fibrosis in our cohort is a limitation that may restrict the generalizability of our findings across the full spectrum of MASLD severity. Although the cohort may overlap with populations previously reported by our group, the present study addresses a distinct research question by specifically investigating the association between short- and medium-chain fatty acids and histological features of MASLD. Finally, the multivariable logistic regression results should be interpreted cautiously because of the sample size and the use of backward variable selection, which may lead to overfitting.

Despite these limitations, our study also has several notable strengths, including the use of liver biopsy for histological characterization and staging of MASLD and the direct quantification of plasma SCFAs and MCFAs using LC-MS. In addition, the simultaneous assessment of multiple SCFAs allowed for a comprehensive evaluation of their differential associations with disease severity.

## 5. Conclusions

In conclusion, patients with MASH showed reduced plasma acetate and increased hexanoate levels compared to patients with no MASLD. Plasma acetate levels were independently associated with MASH and inversely correlated with markers of hepatocellular injury and glycemic control. These findings support a potential association between circulating acetate and MASLD severity and warrant further investigation into the mechanisms underlying this relationship and its potential therapeutic implications.

## Figures and Tables

**Table 1:** Baseline characteristics of the study participants according to histological MASLD status.

*n* = 115		No MASLD	MASL	MASH	*p*
Sex	Men	0 (0%)	15 (36%)	15 (32%)	0.002
	Women	26 (100%)	27 (64%)	32 (68%)	
Age (years)		42 [34–56]	51 [43–56]	49 [42–56]	0.372
Glycemic status	Normal	15 (58%)	10 (24%)	9 (19%)	<0.001
	Prediabetes	9 (34%)	21 (50%)	15 (32%)	
	T2DM	2 (8%)	11 (26%)	23 (49%)	
BMI (kg/m^2^)		42.7 [37.3–45.1]	37.35 [34.6–42.7]	41 [38.5–49.2]	0.621
WC (cm)		119.2 [108.6–127.65] ^a^	125 [120.9–135.4] ^b^	124 [115.6–138.2] ^ab^	0.018
Fat %		47.2 [45.1–49.2] ^a^	45.4 [38.1–47.8] ^b^	44.4 [40.2–47.37] ^b^	0.022
Glucose (mg/dL)		88 [80–95] ^a^	97 [86.1–106.2] ^b^	101.5 [84.3–121.5] ^b^	0.001
HbA1c (%)		5.4 [5–5.9] ^a^	5.85 [5.3–6.2] ^b^	5.8 [5.6–6.7] ^b^	0.001
Insulin (UI/mL)		6.6 [5.5–15] ^a^	12.2 [7.3–18.5] ^a^	13.8 [9.3–25.02] ^b^	0.008
HOMA-IR		1.79 [1.1–3.1] ^a^	3 [2.2–4.4] ^b^	3.3 [2.2–6.8] ^b^	0.001
TG (mg/dL)		121 [112–161] ^a^	171 [147.5–211] ^b^	168 [126.7–213.3] ^b^	0.001
TC (mg/dL)		164 [140–169]	166 [146.2–186.5]	156 [135–175.8]	0.488
HDL-c (mg/dL)		52 [37–61] ^a^	40 [33.3–45.8] ^b^	38 [35–45.75] ^b^	<0.001
LDL-c (mg/dL)		78 [66–98] ^a^	91 [65.3–107.3] ^a^	80 [61.5–94]	0.471
Apo-AI (mg/dL)		153 [125–172] ^a^	134.5 [118.3–150.8] ^b^	132 [121–150.3] ^ab^	0.032
Apo-B (mg/dL)		72 [66–89] ^a^	92 [73.3–102] ^b^	83 [73.3–98.5] ^ab^	0.025
AST (U/L)		15 [13–18] ^a^	18 [14.3–22] ^a^	23 [19–28] ^b^	<0.001
ALT (U/L)		12 [10–19] ^a^	17.5 [11.8–28] ^b^	28 [21.3–40] ^c^	<0.001
GGT (U/L)		14 [10–29.25] ^a^	18 [15.3–20.8] ^a^	23 [16.3–32.8] ^b^	0.018
CK-18 (U/L)		65 [56.1–94.9] ^a^	77.6 [65–108.1] ^a^	106.1 [77.4–212.6] ^b^	0.004

Statistical differences were assessed using the Kruskal–Wallis test for quantitative variables and the chi-square test for categorical variables. Values are presented as median [interquartile range: IQR]. *p* values for sex variable were calculated using chi-square test. For comparisons according to diabetes status, the Kruskal–Wallis test was used, followed by post hoc pairwise comparisons using the Mann–Whitney U test when the overall test was statistically significant. Different superscript letters indicate statistically significant difference at *p* < 0.05 within each row between the groups. Abbreviations: ALT, alanine aminotransferase; AST, aspartate aminotransferase; Apo-A1, apolipoprotein A-1; Apo-B, apolipoprotein B; BMI, body mass index; CK-18, cytokeratin-18; GGT, gamma-glutamyl transferase; HbA1c, glycated hemoglobin; HDL-c, high-density lipoprotein cholesterol; HOMA-IR, homeostatic model assessment of insulin resistance; LDL-c, low-density lipoprotein cholesterol; MASH, metabolic dysfunction–associated steatohepatitis; MASL, metabolic dysfunction–associated steatotic liver; MASLD, metabolic dysfunction-associated steatotic liver disease; T2DM, type 2 diabetes mellitus; TG, triglycerides; TC, total cholesterol; WC, waist circumference.

**Table 2:** Histopathological features of liver biopsies in patients with MASLD.

	MASL	MASH	*p*
SAF score (0/1/2/3/4/5/6/7/8/9)	0/18/16/7/1/0/0/0/0/0	0/0/0/16/10/12/5/2/1/1	<0.001
Steatosis (0/1/2/3)	0/31/11/0	0/25/11/11	<0.001
Hepatocellular ballooning (0/1/2)	24/17/1	0/35/12	<0.001
Lobular inflammation (0/1/2/3)	38/4/0/0	0/35/12/0	<0.001
Fibrosis (0/1/2)	41/1/0	37/9/1	0.013

Values are presented as frequencies. Statistical significance was assessed using Fisher’s exact test, considering *p* < 0.05 to be statistically significant (significant *p* values are represented in bold). Abbreviations: MASH, metabolic dysfunction–associated steatohepatitis; MASL, metabolic dysfunction–associated liver; SAF, steatosis, activity, fibrosis.

**Table 3:** Multivariate logistic regression analysis of factors independently associated with MASH.

Variable	OR (95% CI)	*p*
Constant	0.091	<0.001
Acetate	0.973 (0.947–0.999)	0.040
HOMA-IR	1.176 (0.979–1.412)	0.083
ALT	1.083 (1.035–1.133)	0.001

Values are presented as odds ratios (ORs) and 95% confidence intervals (CIs). *p* values were calculated using multivariate logistic regression with backward Wald selection. Nagelkerke R^2^ = 43.4%; log-likelihood = 53.83. Abbreviations: HOMA-IR, homeostatic model assessment of insulin resistance; ALT, alanine aminotransferase.

## Data Availability

The data that support the findings of this study are available from the corresponding authors upon reasonable request.

## Supplementary Materials

The following supporting information can be downloaded at: https://www.mdpi.com/article/10.3390/nu18213548/s1, Table S1: Univariate logistic regression analysis of factors associated with MASH.

## Abbreviations

The following abbreviations are used in this manuscript:

ALT	Alanine aminotransferase
AMPK	AMP-activated protein kinase
Apo-AI	Apolipoprotein A-I
Apo-B	Apolipoprotein B
AST	Aspartate aminotransferase
BMI	Body mass index
CK-18	Cytokeratin-18
GGT	Gamma-glutamyl transferase
GLP-1	Glucagon-like peptide 1
GPR84	G protein-coupled receptor 84
HbA1c	Glycated hemoglobin
HDLc	High-density lipoprotein cholesterol
IDF	International Diabetes Federation
IQR	Interquartile range
LDLc	Low-density lipoprotein cholesterol
MASH	Metabolic dysfunction-associated steatohepatitis
MASL	Metabolic dysfunction-associated steatotic liver
MASLD	Metabolic dysfunction-associated steatotic liver disease
MCFA	Medium-chain fatty acids
SAF score	Steatosis, Activity, and Fibrosis score
SCFA	Short-chain fatty acids
T2DM	Type 2 diabetes mellitus
TC	Total cholesterol
TG	Triglycerides
VIF	Variance inflation factor
WHO	World Health Organization
