# Supplementary material for: Short- and Medium-Chain Fatty Acid Profiles in Biopsy-Proven Metabolic Dysfunction-Associated Steatotic Liver Disease: A Cross-Sectional Study
Source: Nutrients. 2026 Oct 28;18(21):3548. doi: 10.3390/nu18213548 (PMC13764999; doi:10.3390/nu18213548)
Supplement: Supplementary file 1 [file nutrients-18-03548-s001.zip › nutrients-4604899-supplementary.pdf]

Supplementary Table S1. Univariate logistic regression analysis of factors associated with MASH.

Variable	OR (95% CI)	*p*
Age	1.014 [0.980-1.048]	0.425
Women	0.604 (0.261-1.398)	0.239
BMI	1.005 [0.946-1.067]	0.879
T2DM	4.167 (1.675-10.521)	0.003
HbA1c	1.486 (1.073-2.059)	0.017
HOMA-IR	1.216 (1.070-1.381)	0.003
TG	1 (0.997-1.002)	0.793
HDL-c	0.982 (0.951-1.014)	0.277
Apo-A1	0.993 (0.979-1.007)	0.294
ALT	1.086 (1.045-1.129)	<0.001
Acetate	0.993 (0.985-1.001)	0.084
Hexanoate	1.148 (1-1.317)	0.050

Values are presented as odds ratios (ORs) and 95% confidence intervals (CIs). *P* values were calculated using univariate logistic regression analysis. Abbreviations: T2DM, type 2 diabetes mellitus; HbA1c, glycated hemoglobin; HOMA-IR, homeostatic model assessment of insulin resistance; TG, triglycerides; HDL-c, high-density lipoprotein cholesterol; Apo-A1, apolipoprotein A1; ALT, alanine aminotransferase.
